# Energy-environmental evaluation of conventional and variable rate technology sprayer; application of Life Cycle Assessment

**DOI:** 10.1371/journal.pone.0314911

**Published:** 2025-02-14

**Authors:** Rostam Fathi, Mahmoud Ghasemi-Nejad-Raeini, Saman Abdanan Mehdizadeh, Morteza Taki, Mostafa Mardani Najafabadi

**Affiliations:** 1 Department of Agricultural Machinery and Mechanization Engineering, Agricultural Sciences and Natural Resources University of Khuzestan, Mollasani, Iran; 2 Department of Agricultural Economics, Agricultural Sciences and Natural Resources University of Khuzestan, Mollasani, Iran; ICAR-Indian Agricultural Research Institute, INDIA

## Abstract

The objective of this research was to conduct a comprehensive evaluation of the energy and environmental efficiency of a smart sprayer with a variable rate in orange production. The smart sprayer was developed from a standard orchard sprayer with a fixed rate using different equipment. Energy indicators, encompassing energy efficiency, energy productivity, energy intensity and net energy gain, were calculated. Environmental indicators, including global warming potential, acidification, eutrophication and human toxicity potentials, were estimated using the Life Cycle Assessment (LCA) method in 15 midpoint and 4 endpoint categories. The findings demonstrated that the smart sprayer with a variable rate held a significant advantage over the conventional sprayer with a fixed rate in terms of reducing the pesticide deposition on the trees, lowering pesticide consumption, increasing the energy efficiency and productivity and mitigating the environmental impacts. The maximum pesticide lowering was 46%, achieved at a speed of 1.6 km.hr^-1^. The energy efficiency and productivity changed from 0.594 to 0.650 and from 0.313 to 0.342 kg.MJ^-1^, respectively. The Global Warming Potential (GWP) declined from 422.860 to 407.573 kg CO_2_ eq per ton of orange production. The reduction of chemical pesticide, diesel fuel and agricultural machinery consumption contributed to the decrease in environmental emissions. Consequently, the smart sprayer with a variable rate emerged as a significantly more sustainable and efficient solution for orange production.

## 1. Introduction

The excessive and unregulated use of pesticides poses a serious threat to the health and safety of food consumers. Pesticides contain harmful chemicals that can affect the digestive system and other organs of humans and animals [1]. Without the adoption of technologies and policies to mitigate the environmental impacts of agriculture, the future scenario will be increasingly alarming [2]. New technologies have improved the agricultural production systems in developed countries, and transformed the use of some agricultural inputs, such as fertilizers and chemical pesticides. This has resulted in significant changes in the energy consumption and resource flow for producing different products [3]. Climate change, which is one of the most serious environmental problems in the world, has increased the importance of this issue [4]. To manage the environmental, energy, and economic issues, a comprehensive understanding of the agricultural sector is needed [5]. One of the critical stages in the production of agricultural products is pesticide use with sprayers [6].

Spraying is a common practice to increase the yield and quality of agricultural products [7]. The aim of using chemical pesticides is to deliver an effective and uniform amount of chemicals to the target areas in a safe and timely manner [8]. However, conventional agriculture often uses sprayers with a fixed application rate, which do not consider the actual need for chemicals or the vegetation area and crop density in the field and orchard [9]. This leads to excessive and wasteful use of chemicals, which has negative economic and environmental impacts [10]. Conventional sprayers with a fixed spraying rate have low safety and efficiency, and waste a lot of chemicals by drifting outside the target area [11,12]. The excessive pesticide use increases the production costs and contributed to environmental pollution, as the chemicals contaminate the water, soil, and air [13,14]. Moreover, the chemical droplets can be carried by the wind to sensitive areas, such as populated places [15] or water sources [16], posing a serious threat to human and environmental health. The main drawback of conventional sprayers is that they spray uniformly and indiscriminately, causing harm to the products, people, resources, and the environment. Without adopting new technologies, it is impossible to achieve optimal pesticide application [17].

Many studies have shown that orchard products are excessively sprayed with chemicals, which pollute the air, water, and soil, and damage the resources and the environment [18,19]. Since the canopy structure of trees varies, it is not suitable to use the same amount of chemical pesticides in orchards [16]. However, farmers still use large amounts of chemicals to control pests and diseases in orchards, without considering the differences among orchards and trees in terms of height, canopy width, and leaf area. In traditional sprayers, it is not possible to adjust the amount of pesticide solution based on the canopy, which leads to excessive chemical application on non-target areas [20]. These issues highlight the need for better control over the consumption and distribution of chemicals in conventional sprayers [21]. New technologies can increase the efficiency of sprayers and optimize the use of resources for agricultural production, such as chemical pesticides. By using the best methods, the highest productivity can be achieved [22].

Precision agriculture is a technology that can optimize the pesticide use pesticides by using appropriate equipment for the optimal allocation of inputs. The main goal of this approach is to produce the maximum product with the lowest cost, while preserving the health of the ecosystem and managing the farm effectively [23,24].

Variable rate technology is a key approach in precision agriculture [25]. This technology enables the application equipment to control and detect the information related to the product characteristics and adjust the output of the sprayer accordingly. This technology helps farmers to save pesticides, protect the environment, increase productivity, lower costs, and achieve higher economic profit and sustainable production [26]. Also using Artificial Neural Networks (ANN) and machine learning in orchard sprayers can significantly enhance precision and efficiency [27,28]. These technologies enable sprayers to identify and target specific areas that need treatment, reducing the amount of pesticide used and minimizing environmental impact [29]. By analyzing data from sensors and cameras, ANN can optimize spraying patterns and adjust the dosage in real-time [30]. This leads to healthier crops and reduces the risk of over-spraying [31]. Additionally, machine learning algorithms can predict pest outbreaks, allowing for timely interventions [32].

This study reviewed the recent research on variable rate sprayers for agricultural applications. Variable rate sprayers are devices that can apply a specific amount of chemicals to the target areas, based on the detection of the crop characteristics and the environmental conditions. Variable rate sprayers can reduce the chemical consumption and the environmental pollution, compared to conventional sprayers with a fixed rate. The review summarized the findings of four studies that used variable rate sprayers for different purposes, such as weed control [33], disease detection [34], pest management [35], and tree canopy spraying [36–38]. The studies reported that variable rate sprayers could save between 12% to 73% of the chemical volume, depending on the type of nozzle, the speed of the sprayer, and the crop condition. The studies also evaluated the quality of the chemical deposition and the coverage on the target areas, using water-sensitive papers or other methods. The review concluded that variable rate sprayers were a promising technology for improving the efficiency and sustainability of agricultural production.

This study aimed to develop and evaluate a variable rate sprayer for reducing the energy and environmental impacts of orange production. Variable rate sprayers are devices that can adjust the amount of pesticides applied to the target areas, based on the crop characteristics and the environmental conditions. Previous studies have shown that variable rate sprayers can save pesticides, protect the environment, and increase the efficiency and effectiveness of spraying operations [39–41]. However, there is still a lack of research on the implementation of variable rate spraying in the operational phase and the comprehensive assessment of its energy and environmental impacts [14]. Therefore, this study addressed the existing gaps in the field of variable rate spraying by considering both technical and environmental aspects. The study converted a turboliner sprayer (conventional sprayer) into a variable rate sprayer using the necessary equipment. The variable rate sprayer was developed and tested for its technical performance and its chemical saving potential. The energy and environmental impacts of orange production using the conventional sprayer and the variable rate sprayer were compared and analyzed. **Table 1** shows some of the indicators used in the study and their sources.

**Table 1:** Some study indicators in the conducted research.

Researcher	Paper	Quantitative performance	environmental impacts	Orchard sprayer	Agricultural sprayer	Fixed rate sprayer	Variable rate technology sprayer
[38]	Sonar sensing predicated automatic spraying technology for orchards	*	-	*	-	-	*
[16]	Spray pesticide applications in Mediterranean citrus orchards: Canopy deposition and off-target losses	*	-	*	-	*	-
[42]	Development of a LiDAR-guided section-based tree canopy density measurement system for precision spray applications	*		*			*
[20]	Chemical footprint of pesticides used in citrus orchards based on canopy deposition and off-target losses	*	*	*	-	*	-
[43]	Modeling the carbon footprint of fresh produce: effects of transportation, localness, and seasonality on US orange markets	-	*	*	-	*	-
[44]	Multiyear life energy and life cycle assessment of orange production in Iran	-	*	*	-	*	-
[45]	Organic versus conventional citrus. Impact assessment and variability analysis in the Comunitat Valenciana (Spain)	-	*	*	-	*	-
Current Study	**Energy-environmental Evaluation of Conventional and Variable Rate Technology Sprayer; Application of Life Cycle Assessment**	*	*	*	**-**	*	*

## 2. Materials and methods

### 2.1. Development of conventional sprayer to variable rate sprayer

In this study, a conventional sprayer was transformed into a variable rate sprayer using a turboliner sprayer and the impact of variable rate spraying technology (**Table 2**) on the energy and environmental aspects of orange cultivation was evaluated from 10 October 2023 to 15 October 2023.

**Table 2:** Characteristics of the orchard sprayer used in this research.

Title	Property
Type of sprayer	Orchard and farm turboliner sprayer
Manufacturer	CAFFINI (Italy)
Tank volume (Liter)	800
Pump type	Piston
Maximum pressure (bar)	Adjustable and maximum pressure 35 bar
Number of nozzles	10
Nozzle type	Conical
Maximum spraying radius (meters)	20
Type of tank	Polyethylene plastic
Agitator of the Chemical inside the Sprayer tank	yes
Type of connection to the tractor	Mounted and PTO

In general, the development of the variable rate sprayer consisted of two stages:

1. Canopy detection Phase
2. The decision-making Phase and controlling the spraying rate

**Fig 1:**
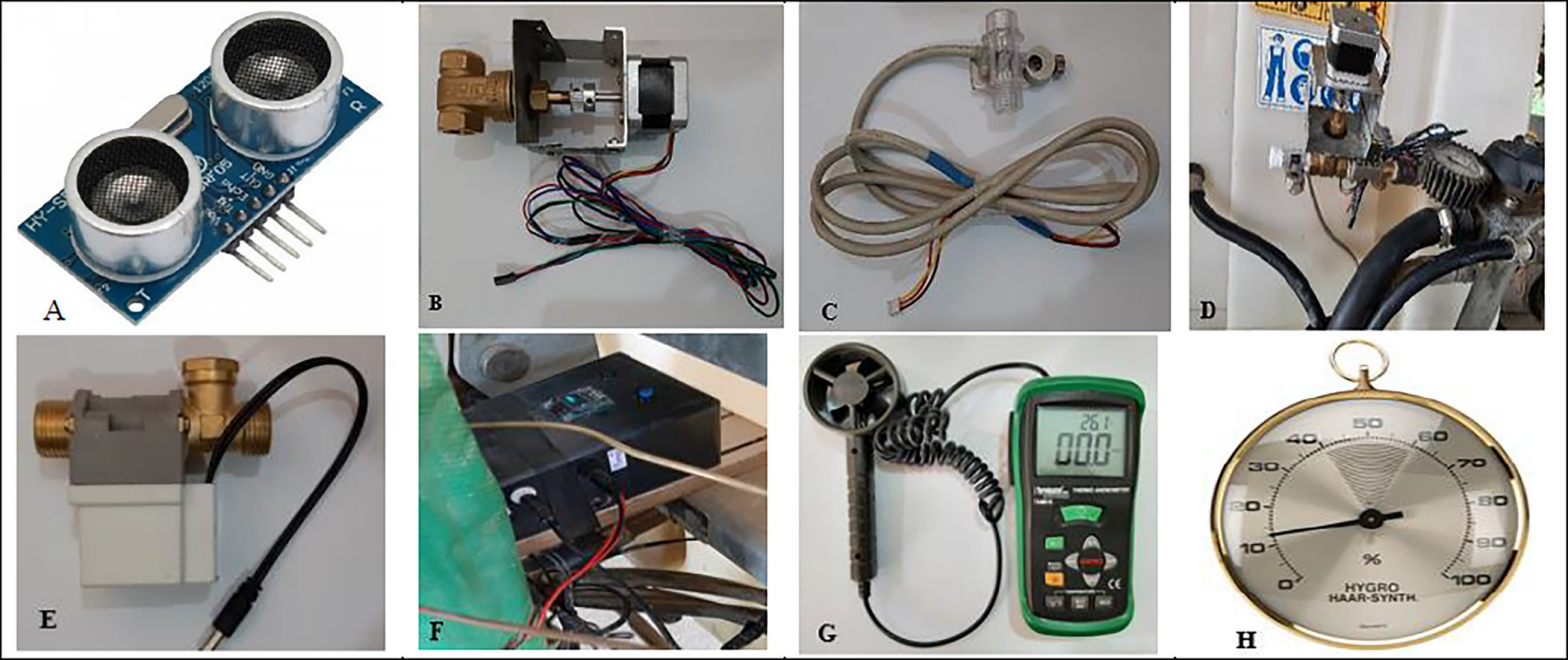
A: Ultrasonic sensor B: Variable rate valve C: Flowmeter (YF-S201C Water flow sensor) D: Installing a variable rate valve and flowmeter on the sprayer E: ON and OFF solenoid valve (electric solenoid valve 12v 24V 220V) F: Installing the electronic board on the sprayer G: Anemometer and thermometer H: Hygrometer.

**Fig 1** shows some of the equipment used in the development of the fixed-rate sprayer to variable-rate sprayer and in the field tests.

### 2.2. Canopy detection phase

To avoid wasteful use of chemicals, a detection system using image processing was implemented for the identification of the canopy of the tree, thereby establishing the basis for the application of a variable chemical for spraying. The canopy detection system was further utilized to discontinue the spraying flow in the vacant spaces between the trees, where the absence of the canopy negates the necessity for spraying.

To prevent the continuous operation of the variable rate valve, an on-off solenoid valve was incorporated into the spraying flow. The activation of this valve was governed by an ultrasonic sensor which was affixed to a specially fabricated base on the sprayer body. A certain distance (one meter) was set in software for the ultrasonic sensor to detect the tree canopy, implying that the on-off solenoid valve would cut off the entire flow of the output solution if no canopy was detected within one meter in front of the ultrasonic sensor. The operation of the on-off solenoid valve was controlled by electronic board based on the distance detected by the ultrasonic sensor.

In this research, to prevent the suboptimal consumption of pesticides solution, a canopy volume detection system was utilized through image processing, and the canopy volume of the tree was used as the basis for applying the variable rate of pesticides solution. Another application of the canopy volume detection system was to halt the spraying flow in the empty spaces between the trees, which did not require spraying due to the absence of the canopy. A color digital camera was used to take pictures and the structure-from-motion method was used to reconstruct 3D information [46]. MATLAB software was used for 3D reconstruction. In this method, the three main phase that were followed to produce 3D point clouds were:

- Feature matching: where the relationship between different points in the images was calculated;
- Camera estimation: using previous connections, camera parameters and locations were estimated for each image;
- Dense reconstruction:

Camera parameters were used to display two-dimensional image points in the corresponding three-dimensional locations.

The relationship between two-dimensional image points and three-dimensional locations was obtained through the pinhole camera model method. In this model, x was a representation of a three-dimensional point in homogeneous coordinates (a four-dimensional vector) and p was a two-dimensional image display of this point in a three-dimensional vector in homogeneous coordinates. The relationship between them is (Eq 1) [46]:

$$P=C_{i}\times X$$ (1)

where, Ci is the camera 4 × 3 matrix, which shows the internal camera parameters (K matrix) and external parameters ([RiTi] matrix) of camera I was calculated by [46]:

$$C_{i}=K[R_{i}T_{i}]$$ (2)

In this research, since all the images were taken with the same camera, the parameters of the primary camera were shared among all the images. However, the internal parameters varied for each image. Therefore, rotation matrices RiRi and translation vectors TiTi were defined for each image. By knowing the internal parameters of the camera (matrix KK) as well as the position and orientation of all images (matrix [Ri,Ti][Ri,Ti]), two-dimensional image recognition was performed on the three-dimensional point cloud (Eqs 1 and 2). RGB images were captured from the side of the trees using a camera connected to the sprayer. The calculated volume (processed by the computer) was then sent to the control circuit as input, and the output in the form of a canopy volume model ultimately determined the required amount of solution and the spraying rate of the sprayer. In general, the image processing module was responsible for detecting and calculating the canopy volume of the tree, and the canopy volume determined by image processing served as the basis for establishing the spraying rate of the pesticide solution.

### 2.3. Spraying experiment with variable rate technology in the orchard

In this study, all the experiments were conducted in two modes of fixed (conventional sprayer) and variable rate spraying and at three speeds: low (1.6 km.hr^-1^), medium (3.2 km.hr^-1^) and high (4.8 km.hr^-1^) in orange orchards with three repetitions and measured the performance indicators of spraying. Also, all the other parameters such as fan speed and spraying pressure were constant in this research for all experiments. Wind speed, temperature and air humidity during the experiments were recorded. In this research, Harhygrometer E-Precision hygrometer ((Reading accuracy: ±5%. Diameter: 100mm. made in Germany) was used for showing humidity in the range of 0 to 100% and at a temperature of -35 to +65 degrees Celsius. Also, TAM 618 thermometer and anemometer (Wind speedometer model DT-618 made by CEM company. Made in China) were applied to measure temperature and wind speed.

### 2.4. Determining the leaf surface of the tested trees

In order to determine the amount of leaf area of the investigated trees in the spraying operation, two indices of leaf area density (LAD) and tree canopy were measured by considering the area of two leaf tops [20]:

$$S_{T}=2\times \mathrm{LAD}\times V_{T}$$ (3)

where:

**LAD** is leaf surface density (m2ofleavesm3canopy).

**V**_**T**_ is the average apparent volume of the tree canopy (m2tree).

For this purpose, a cube measuring 50×50×50 cm was fabricated, and leaves from were various parts of the tree were detached, collected and weighed. Then, the leaf area of the tree was computed using leaf weight ratio per canopy volume unit.

To obtain the ratio of leaf surface to its weight, a set of 10 leaves was weighed using a digital scale, and the surface area of these leaves was determined through image processing. Ultimately, the Leaf Surface Density (LSD) area was computed by using the two derived ratios: the leaf weight (LW) per unit of canopy volume (CV) (Eqs 4, 5) and the leaf area to its weight [20]:

$$\mathrm{TLS}=\frac{\mathrm{LW}}{\mathrm{CV}}$$ (4)

Where, LW is weight of leaves (gram per leaf) and CV is volume of tree canopy (m^3^ per tree): [20]:

$$\mathrm{LSD}=\frac{LAD}{\mathrm{LW}}$$ (5)

Where, LAD is leaf surface density (m^3^ of leaves per m^3^ of canopy) and LW is leaf weight (grams per leaf).

To compute the apparent volume of the trees, the canopies were postulated to be oval in shape, and their volume was accordingly calculated using (Eq 6). This involved capturing one image perpendicular to the planting line and another image along the planting line. The average diameter derived from these two images, after segregating the tree’s image from the background, was incorporated into (Eq 6) to ascertain the volume of the plant [20]:

$$V_{T}=\frac{1}{6}\times \pi \times d_{ac-r}\times d_{al-r}$$ (6)

**Fig 2:**
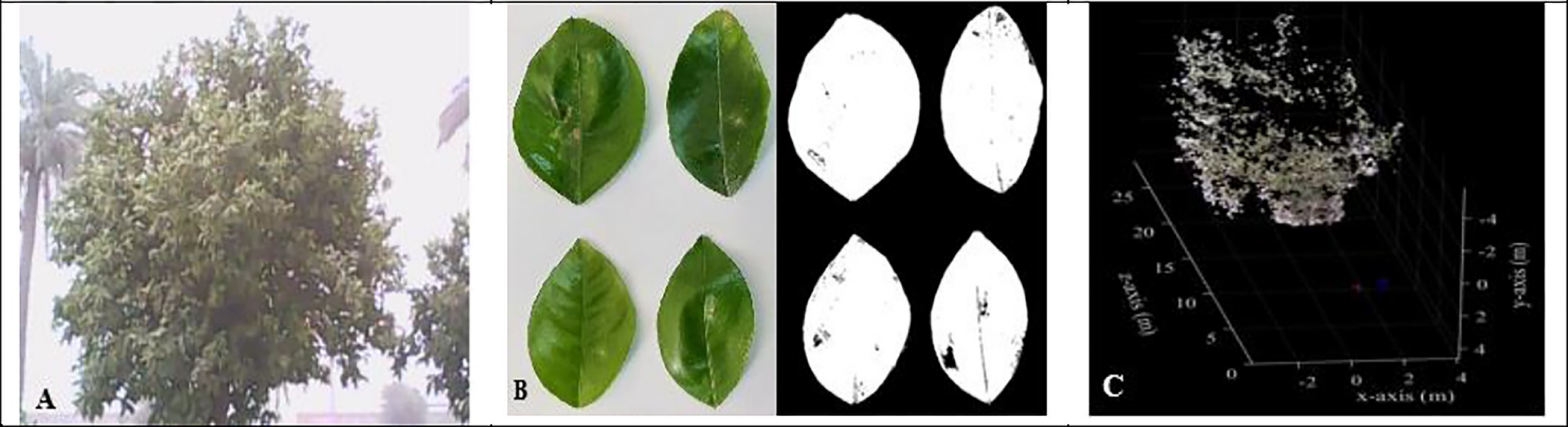
A: Image of the tree by a digital camera B: Determining the leaf area C: Calculating the canopy volume.

In the given equation, d_ac-r_ represents the mean diameter of the tree canopy perpendicular to the row, while d_al-r_ denotes the mean diameter of the canopy along the row of trees. The variable h stands for the height of the tree canopy. **Fig 2** shows the procedure of calculating the volume of the canopy.

### 2.5. Comparison and evaluation of the amount of pesticide spraying on the canopy in two modes of spraying (Conventional spraying and variable rate technology spraying)

**Fig 3:**
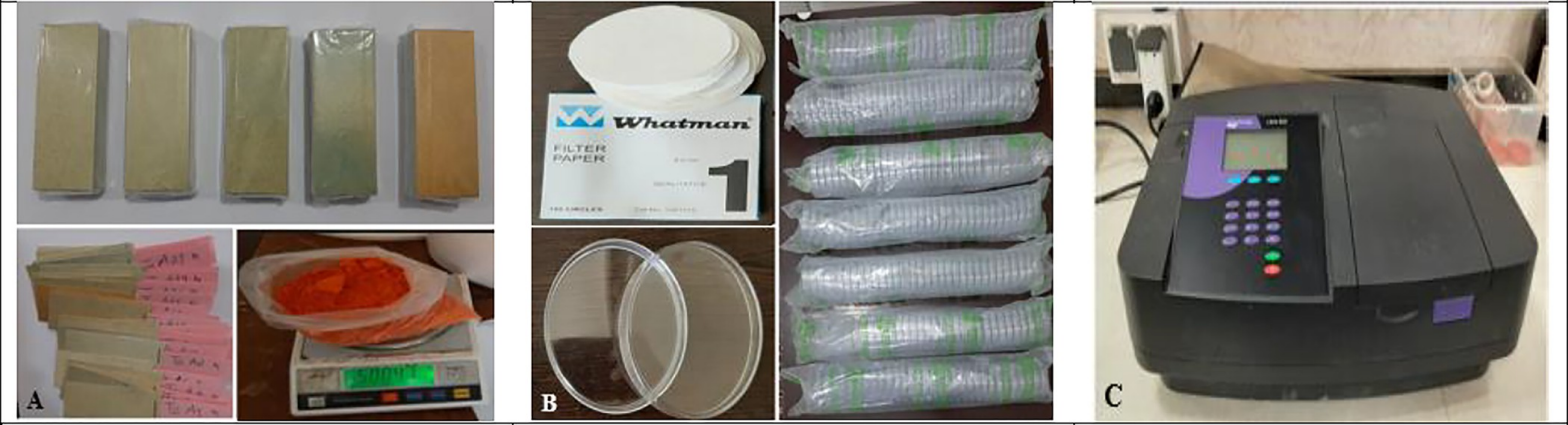
A: Papers sensitive to water and tartrazine dye B: Whatman filter paper and Petri dish C: Biochrom Libra S22 UV-Vis optical spectrometer.

For the spraying experiments, a solution of water and yellow tartrazine dye (with chemical formula: C16H9N4NA3O9S2) was used to quantify the consumption rate and the concentration of chemicals on the canopy as well as the space outside the canopy. This dye is a type of edible colorant that dissolves in water at the rate of 5–6 grams per liter [47]. **Fig 3** shows some of the materials and equipment used in the process of determining the amount of spray deposition.

### 2.6. Evaluation the energy indicators

For the computation of energy indicators, data pertaining to the inputs consumed in one hectare of an orange orchard was collected. Subsequently, the energy of each input was derived from the table of equivalents (**Table 3**), and **Table 4** was utilized to calculate the input energy of each input [48].

**Table 3:** Energy equivalent of inputs and output in orange production.

Item	Unit	Energy equivalent (MJ unit^-1^)	Reference
**A. Inputs**			
1. Human labor	hr	1.96	[49]
2. Agricultural machinery	h	62.7	[50]
3. Diesel fuel	L	56.31	[51]
4. Chemical fertilizers	kg		
Nitrogen (N)	kg	66.14	[51]
Phosphate (P_2_O5)	kg	12.44	[51]
Potassium (K_2_O)	kg	11.15	[52]
5. Chemical pesticides	kg		
Pesticide	kg	199	[53]
Herbicide	kg	238	[53]
Fungicide	kg	92	[53]
6. Transportation	T.km	62.7	[54]
7. Electricity	KW.h	11.93	[55]
8. Water for irrigation	m^3^	1.03	[56]
**B. Output**			
Orange	kg	1.9	[55]

**Table 4:** Equations used in the estimation of input and output energy in orange production.

Inputs energy	Equation	parameters
S_E_ = Seed energy (MJha^-1^)	$S_{E}=\frac{S_{Q}+S_{C}}{A}$	S_Q_ = Quantity of seed used (kg) S_C_ = Energy conversion factor (MJkg^-1^) A = Size of farm (ha)
F_E_ = Diesel fuel energy (MJha^-1^)	$F_{E}=\frac{F_{C}+f_{C}}{A}$	F_C_ = Fuel consumed (L) f_C_ = Energy conversion factor (MJL^-1^) A = Size of farm (ha)
H_E_ = Human labor energy (MJha^-1^)	$H_{E}=\frac{H+l_{C}}{A}$	H *=* Working duration(h) l_C_ = Energy conversion factor (MJh^-1^) A = Size of farm (ha)
CF_E_ = Chemical fertilizer energy (MJha^-1^)	${\mathrm{CF}}_{E}=\frac{Q(N,P_{2}O_{5},\mathrm{Mn})\times N(\mathrm{CN},P_{2}O_{5},\mathrm{Mn}}{A}$	Q(N,P_2_P_5_, Mn) = Quantity of N, *P*_2_*O*_5_ and MnSO_4_ (kg) N_C_ = Nutrient (N, P_2_O_5_, Mn) energy conversion factor (MJkg^-1^) A = Size of farm (ha)
AM_E_ = Agri-machinery energy input (MJ ha^-1^)	${\mathrm{AM}}_{E}=\frac{W}{L\times A}\times C_{F}\times T$	C_F_ = Energy conversion factor (MJkg^-1^) W = Weight of agri-machinery (kg) L = Useful life of agri-machinery (h) T = Working time (h) A = Size of farm (ha)
E_E_ = electricity energy input (Kwh ha^-1^)	$E_{E}=\frac{g\times \rho \times H\times ɸ}{\varepsilon_{1}\varepsilon_{2}}$	g = gGravitational acceleration (m/S^2^) ρ = Water density (kg/m^3^) H = Total dynamic well head (h) ɸ = Water flow rate(m^3^/ha) ε_1_ = Pumping efficiency varying between 0.7 and 0.9 ε_2_ = Efficiency of energy and power (0.18–0.22 for electro pump and 0.25–0.30 for diesel)

After calculating the energy inputs, using specific relationships, energy indices was calculated (**Table 5**) [57]. These indicators provide researchers and managers of production units with the possibility to examine product production systems in more detail in terms of energy efficiency [58].

**Table 5:** Energy indices in cropping system of orange production.

Index	Unit	Equation	Reference
Energy use efficiency or energy ratio (ER)	-	$\frac{\mathrm{Output}\;\mathrm{energy}{(\mathrm{MJ}\;\mathrm{ha}}^{‐1})}{\mathrm{Total}\;\mathrm{input}\;\mathrm{energy}{(\mathrm{MJ}\;\mathrm{ha}}^{‐1})}$	[59]
Energy productivity (EP)	Kg MJ^-1^	$\frac{\mathrm{Yield}{(\mathrm{Kg}\;\mathrm{ha}}^{‐1})}{\mathrm{Total}\;\mathrm{input}\;\mathrm{energy}{(\mathrm{MJ}\;\mathrm{ha}}^{‐1})}$	
Specific Energy (SE)	MJ Kg^-1^	$\frac{\mathrm{Total}\;\mathrm{input}\;\mathrm{energy}{(\mathrm{MJ}\;\mathrm{ha}}^{‐1})}{\mathrm{Yield}{(\mathrm{Kg}\;\mathrm{ha}}^{‐1})}$	
Net Energy Gain (NEG)	MJ ha^-1^	Output energy (MJ ha^-1^)-Total input energy(MJ ha^-1^)	
Direct energy (DE)	MJ	Human labor + diesel fuel + electricity + seed	
Indirect energy (IDE)	MJ	Machinery + chemical fertilizers + FYM + biocides	
Renewable energy (RE)	MJ	Human labor + FYM + seed	
Non-renewable energy (NRE)	MJ	Machinery + chemical fertilizers + diesel fuel + biocides + electricity	

### 2.7. Evaluation of environmental impacts

#### 2.7.1. Definition of life cycle assessment

**Fig 4:**
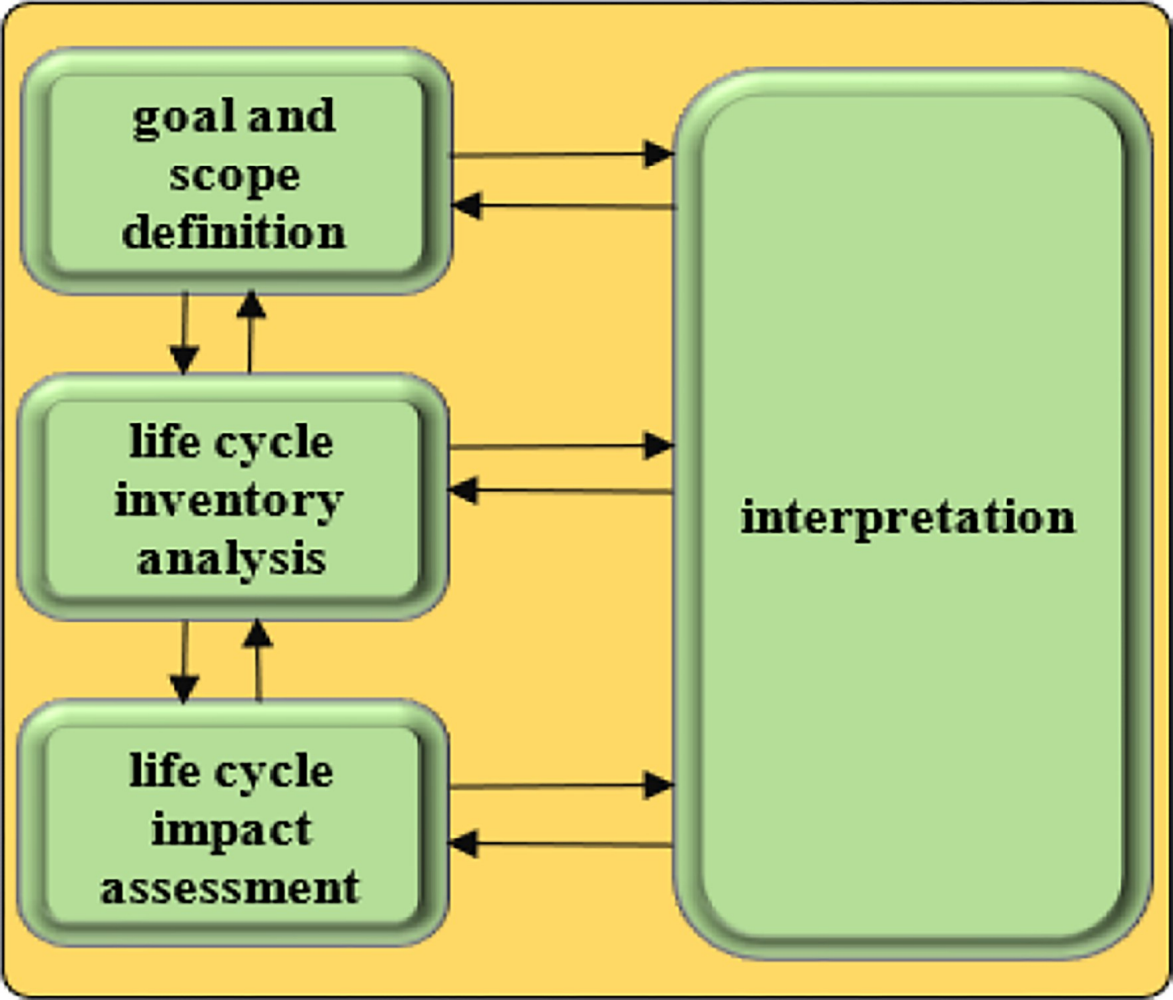
Life Cycle Assessment framework.

Life Cycle Assessment (LCA) is an approach that assesses the inputs, outputs and environmental effects of all stages of a product’s life or production process [60]. This approach has four general phases: goal and scope definition, life cycle inventory analysis (LCI), Life Cycle Impact Assessment (LCIA) and interpretation of results [51]. In the present study, FU (Functional unit) as one ton of oranges and the system boundary included all inputs and agronomic operations in the orange production farm were defined [61]. **Fig 4** shows the phases of Life Cycle Assessment.

A questionnaire was used to collect the required data and the equivalent of the inputs used was obtained from the Ecoinvent database (EcoInvent version 3.8) [62]. The Impact 2002 model was used in Simapro software for this research. This method classified and identified environmental emissions under four end categories and 15 intermediate categories. The data collection period in this study was from the start of the first irrigation phase to the end of the harvest in the orchard. In other words, the cradle-to-gate method was the basis of this research.

The evaluation unit for this study was set at one hectare for calculating energy indicators, while the functional unit was defined as one ton of citrus fruits for assessing environmental impacts. The primary objective of this research was to compare the environmental effects and interpret the inputs and outputs from spraying operations across two scenarios: the use of a variable rate sprayer system versus an orchard constant rate sprayer. This analysis was conducted using SimaPro software, following a structured four-phase approach [60]:

- Selection and Classification of Impact Categories
- Characterization of Effects
- Normalization
- Weighting

**Fig 5:**
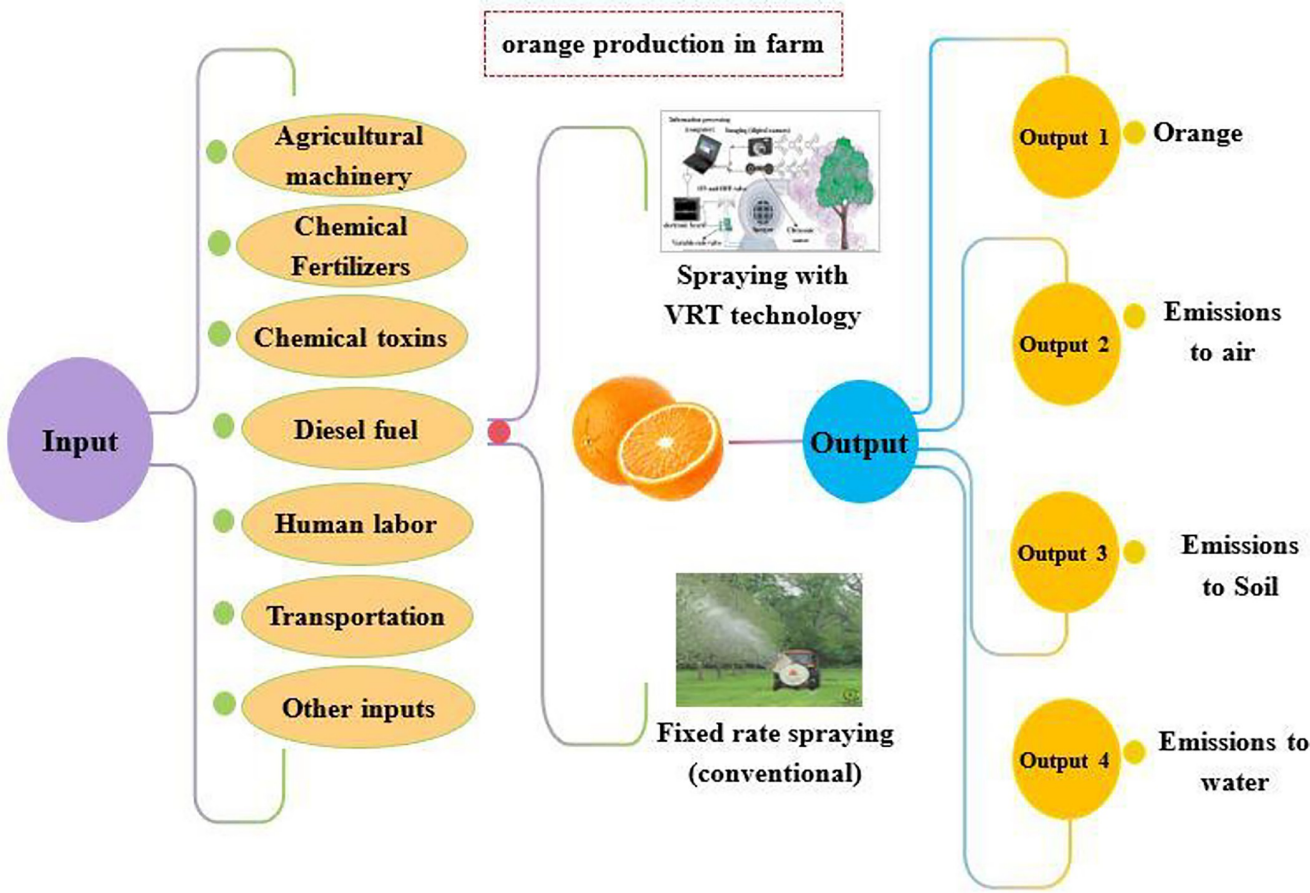
The system boundary of orange production.

In this phase of the research, we evaluated the environmental impacts associated with various inputs used in spraying operations, including chemical pesticides, agricultural machinery, transportation for supplying spraying water via tractor, and fuel consumption. This evaluation was carried out for both fixed-rate spraying and variable-rate spraying scenarios. The findings from this study are expected to provide valuable insights into the efficiency and sustainability of pesticide application methods in citrus production. By analyzing the differences in environmental impacts between the two spraying techniques, we aim to identify best practices that can lead to reduced chemical usage and lower overall environmental footprints. This research not only contributes to the understanding of agricultural sustainability but also supports the development of more effective pest management strategies that align with environmental conservation goals. The boundary of the orange production system in this study is shown in **Fig 5**.

**Fig 6:**
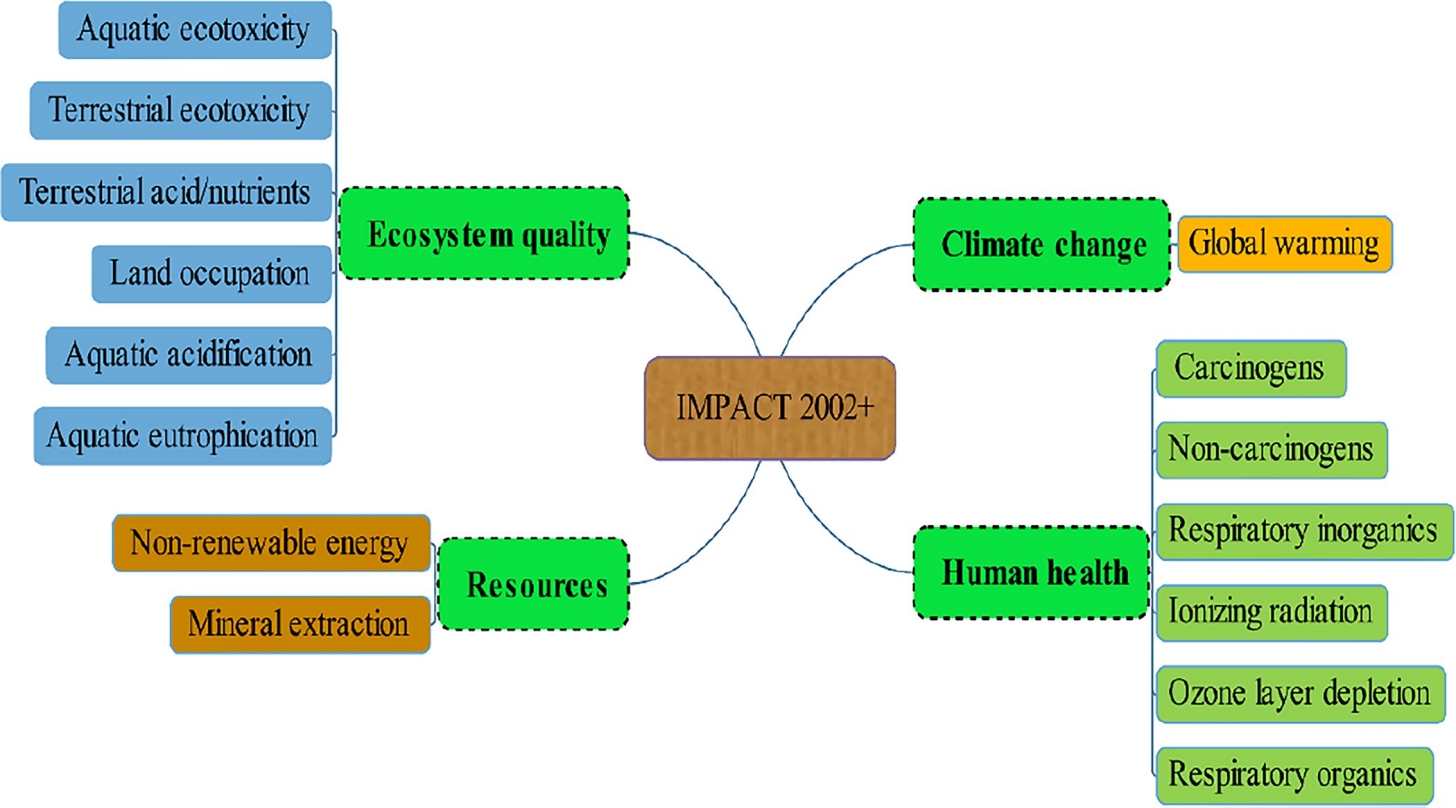
Distribution of 15 midpoints based on IMPACT 2002+ method.

In this research, the IMPACT 2002+ method was used to evaluate the environmental Impact Assessment. **Fig 6** shows the distribution of 15 middle and 4 final categories in IMPACT 2002+ method.

## 3. Results and discussion

### 3.1. The amount of total consumption of pesticides solution in different modes of spraying

**Table 6** shows the total consumption of pesticides per hectare, as well as their settling on trees, ground and air release in different spraying modes. The results showed that the variable rate spraying mode at a speed of 1.6 km.hr^-1^ (1952 L.ha^-1^) had the lowest amount of pesticides consumption and the constant rate spraying mode at a speed of 1.6 km.hr^-1^ (4014 L.ha^-1^) had the highest amount of pesticides consumption. The application of variable rate technology in orchard spraying resulted in the maximum amount of savings at the movement speed of 1.6 km.hr^-1^ (46%).

The average amount of pesticides deposition was higher in the fixed rate spraying than the variable rate spraying for all treatments.

**Table 6:** The total amount of pesticides consumption per hectare, sitting on trees, ground and air release in different modes of spraying.

Title	T 1	T 2	T 3	T 4	T 5	T 6
Amount of sediment on trees (L.ha^-1^)	1527	1387	1303	2121	1983	1646
Amount of settling on the ground (L.ha^-1^)	546	616	492	1754	1663	1622
Emission amount in the air (L.ha^-1^)	113	105	156	139	131	241
Sum of total deposition on trees, ground and air emissions (L.ha^-1^)	2185	2107	1952	4014	3777	3509
Reduction in consumption compared to the fixed rate (L)	1829	1670	1557			
Savings percentage	46%	44%	44%			

In the case of variable rate spraying and at low speed (1.6 km.hr^-1^), the total amount of pesticide spraying in a certain distance is higher than when the speed of movement is higher. On the other hand, when the spraying speed is low (1.6 km.hr^-1^), The precision of the variable rate system in cutting and connecting the spraying flow is very high, and this caused the maximum amount of poison to settle on the tree and the minimum amount of pesticide to settle on the ground. When the spraying speed increased, there was some delay in stopping the spraying flow, which caused some spraying of pesticide on the ground before and after the tree. For this reason, in the second treatment (spraying at a speed of 3.2 km.hr^-1^), the amount of pesticide on the ground increased compared to the first treatment. In the third treatment (spraying at a speed of 4.8 km.hr^-1^), since the speed of the sprayer was higher than in the first and second treatments, the total amount of pesticide sprayed was also less, because there was little time for the sprayer to pass through the specified path. For this reason, both the amount of pesticide on the tree and the amount of pesticide on the ground decreased.

**Table 7** shows the variance analysis of the total pesticide’s consumption in different spraying modes. The variance analysis for the pesticides deposition on trees, land, air release and total pesticides consumption per hectare revealed a significant difference between the variable rate sprayer and the fixed rate sprayer modes. The type of sprayer and movement speed had a significant impact on the total pesticide’s consumption, but their interaction effect was not significant (p<0.05).

**Table 7:** The results of the ANOVA test for the total amount of pesticides consumption in different modes of spraying.

Source of variation	df	Mean Square	F	Sig.
Speed	2	274440.056	7.230	0.009**
Type of sprayer	1	13629420.500	359.046	0.001**
Speed × Type of sprayer	2	56405.167	1.486	0.265^ns^
Error	12	37960.111		
Coefficient of Variation (CV)	0.32			

ns: None significant, *Significant at the probability level of 5%, **Significant at the probability level of 1%.

In a research, researchers showed that increasing the spraying speed reduces the pesticide solution used, and increasing the spraying pressure for all types of nozzles increases it in both machine vision and non-vision modes [63]. Using the machine vision technique, the N4 type nozzle had the maximum saving percentage of the spraying solution (57.57%) at 400 kPa spraying pressure and 0.27 ms^-1^ movement speed compared to the non-vision mode. Using the machine vision technique, the N1 type nozzle had the minimum saving percentage of the spraying solution (47.82%) at 250 kPa spraying pressure and 1.12 ms^-1^ movement speed compared to the non-vision mode.

The results of the current research showed that in the variable rate spraying, compared to the fixed rate spraying mode, the amount of pesticide consumption and the amount of environmental impacts decreased. In addition, the value of energy efficiency index and energy productivity increased and energy intensity decreased. The results showed that by reducing the consumption of pesticide in the variable rate spraying mode, the cost of purchasing pesticide was also reduced. The results of the present study showed that the use of variable rate spraying can play an important role in achieving sustainable agriculture by improving energy indicators, reducing environmental impacts, reducing costs, and increasing production profitability. Since achieving optimal consumption of pesticides using variable rate spraying technology is not affected by the geographical region, the results of this research can be useful in other countries in addition to Iran. In other words, the application of variable rate technology in different orchards can lead to the improvement of energy, environmental and energy indicators in all regions of the world. Since different methods for varying the rate of sprayers have been proposed and implemented in some areas, the type of equipment used in different countries can have limitations according to technical and economic conditions. Therefore, the principle of variable rate, regardless of the type of equipment used in the process of making variable rate sprayers, can play an effective role in increasing the productivity and sustainability of agricultural production.

### 3.2. Evaluation results of energy indicators

**Fig 7:**
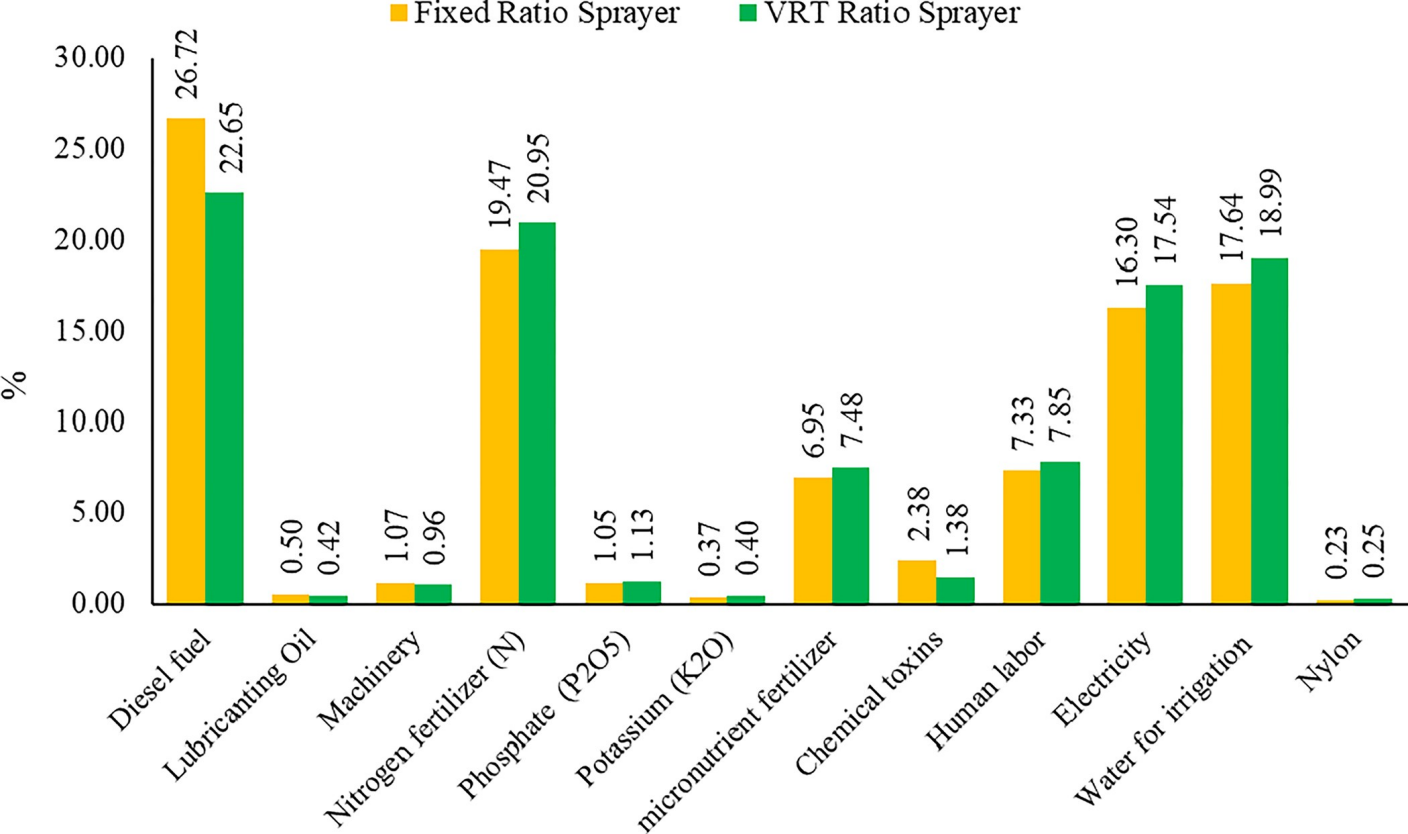
Contribution of inputs in the orange production process under two scenarios of using fixed rate and variable rate sprayer.

The energy of different inputs and obtained their contribution to the total input energy were calculated. The contribution of each input in the orange production process in two scenarios of fixed rate and variable rate spraying was shown in **Fig 7**.

**Fig 8:**
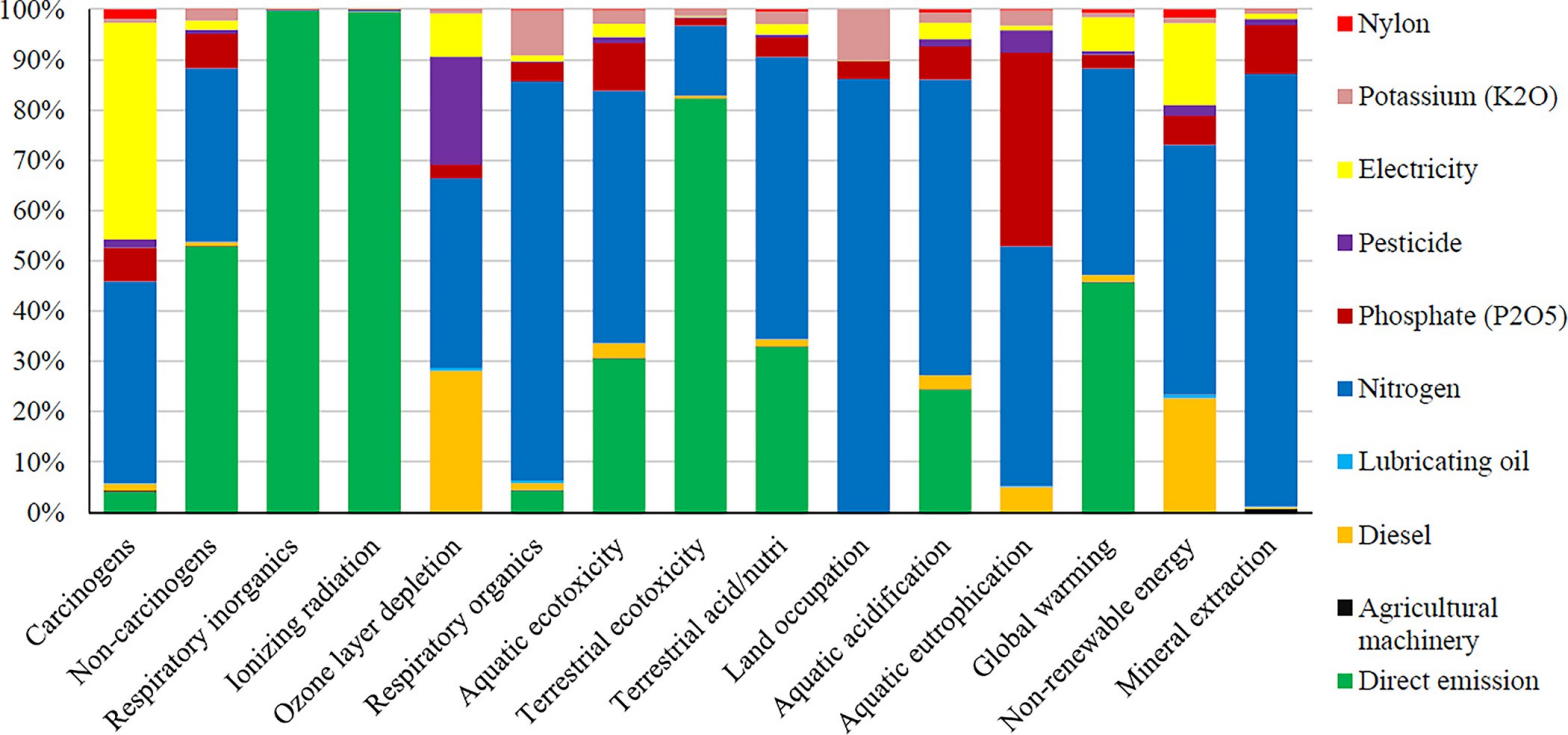
The results of the environmental impact Assessment in 15 intermediate impacts categories for using a fixed rate sprayer.

As shown in **Table 8,** diesel fuel consumption was the most substantial input in the orange production process per hectare. The consumption of this input was 274.38 liters (26.72%) for constant rate spraying and 216.11 liters (22.65%) for variable rate spraying. Variable rate spraying reduced the fuel consumption by decreasing the tractor transportation needed to supply the required water. The consumption of different chemical pesticides was 8.23 liters (2.38%) for fixed rate spraying and 4.44 liters (1.38%) for variable rate spraying. Variable rate spraying saved 46% of pesticide consumption compared to fixed rate spraying. Variable rate spraying also reduced the consumption of diesel fuel, chemical pesticide and oil used in tractors. **Fig 8** shows the energy consumption percentage of each input in the orange production process per hectare for the two scenarios.

**Table 8:** Share of different inputs and output energy in fixed and VRT ratio sprayer.

Input-output inputs	Unit	Fixed Ratio Sprayer		VRT Sprayer	
		Amount (ha)	Energy (MJha^-1^)	Amount (ha)	Energy (MJha^-1^)
Diesel fuel	L	274.38	15450.06	216.11	12169.10
Lubricating Oil	L	6.02	287.61	4.74	226.34
Machinery	hr	1026.90	617.07	1003.59	515.04
Nitrogen fertilizer (N)	kg	370.00	11257.03	370.00	11257.03
Phosphate (P_2_O_5_)	kg	106.25	608.01	106.25	608.01
Potassium (K_2_O)	kg	53.75	215.75	53.75	215.75
Micronutrient fertilizer	L	33.50	4020.00	33.50	4020.00
Chemical pesticides	L	8.23	1374.25	4.44	742.10
Human labor	hr	2162.75	4238.99	2151.10	4216.15
Electricity	Kwh	790.00	9424.70	790.00	9424.70
Water for irrigation	M^3^	10000.00	10200.00	10000.00	10200.00
Nylon	kg	7.38	132.09	7.38	132.09
Orange	kg	18375	34912.50	18375	34912.50

The comparison of energy indicators in two spraying scenarios showed that changing the type of sprayer from fixed rate to variable rate improved the energy indicators (**Table 9**). The results indicated that variable rate spraying reduced the input energy in the orange production process compared to fixed rate spraying. Energy efficiency increased from 0.594 to 0.650 and energy productivity increased from 0.313 to 0.342 kgMJ^-1^. Energy intensity decreased from 3.20 to 2.92 MJkg^-1^. The main factors for improving energy indicators were lower consumption of diesel fuel, chemical pesticides and water, and less tractor transportation to supply water.

**Table 9:** Energy indices in orange production.

Index	Unit	Fixed Rate sprayer	Variable Rate sprayer
ER	-	0.594	0.650
EP	Kg MJ^-1^	0.313	0.342
SE	MJ Kg^-1^	3.20	2.92
NEG	MJ ha^-1^	-23829.32	-18813.80
DE	MJ	39604.89	36236.29
IDE	MJ	19136.93	17490.01
RE	MJ	4242.52	4216.15
NRE	MJ	54499.30	49510.15
Total input energy	MJ	58741.82	53726.30
Total output energy	MJ	34912.50	34912.50

### 3.3. Evaluation results of environmental indicators

In this study, present the environmental impact assessment results in 15 intermediate and four final impact categories in this section. **Fig 8** shows the environmental indicators for producing one ton of oranges using a fixes rate sprayer in the orchard.

This research estimates the global warming potential to be 422.85 kg of carbon dioxide equivalent (kg CO_2_ eq) per ton of oranges produced in Dezful region.

Nitrogen fertilizer had the most environmental impacts across most impact categories. Direct emissions from the inputs, depicted in the aforementioned graph as direct emissions, had the second-highest environmental impacts. Both direct emissions and nitrogen fertilizer had the most substantial effect on global warming.

**Fig 9:**
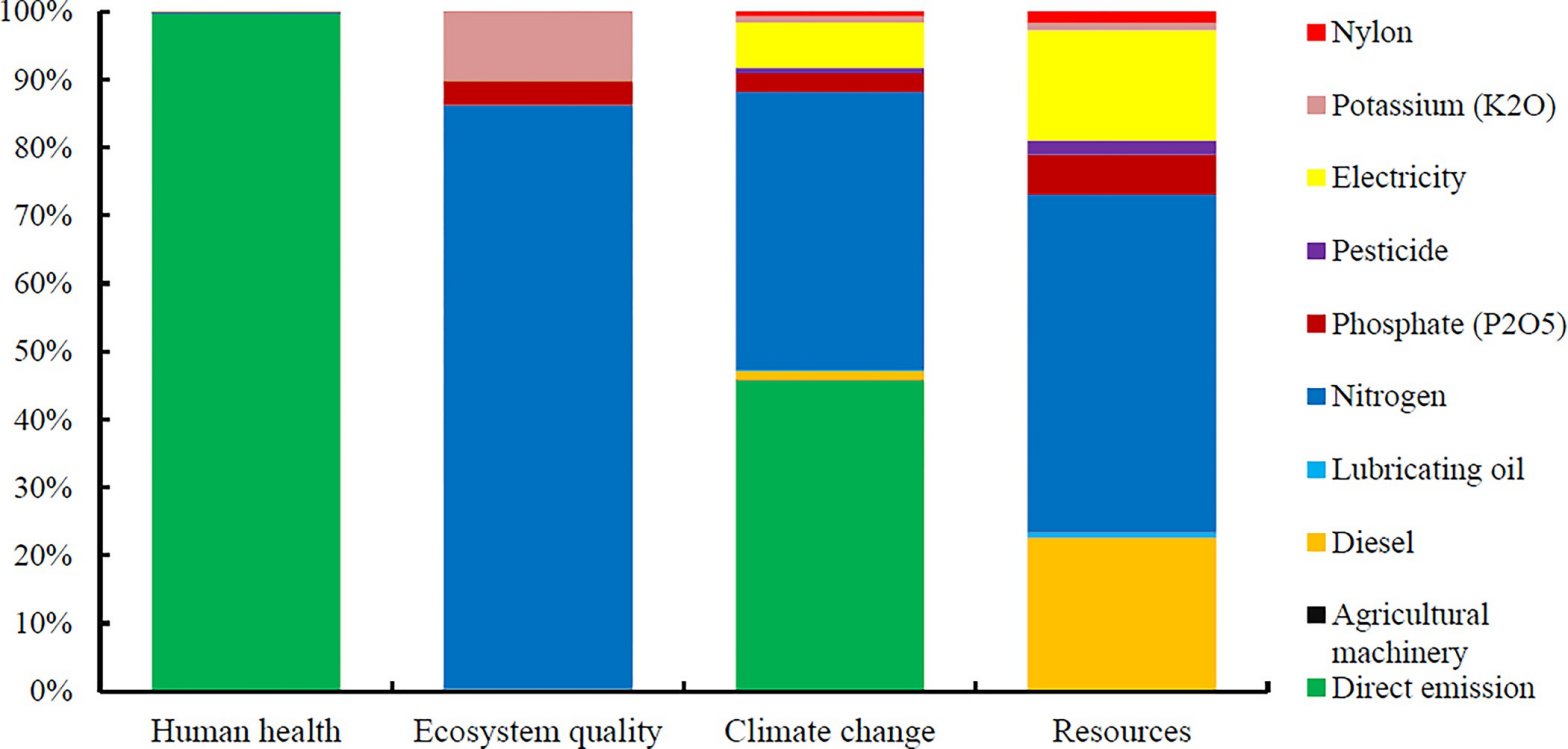
The results of environmental impact assessment in 4 final impacts categories for using fixed rate sprayer in.

The environmental performance evaluation of orange production in Mexico using the life cycle assessment (LCA) approach revealed that the production and use of chemical fertilizers are the primary contributors to environmental impacts associated with orange cultivation [64]. Another study demonstrated that nitrogen fertilizers can significantly reduce abiotic and human toxicity while simultaneously being major contributors to global warming and photochemical oxidation within the context of orange production [44]. Additionally, the study indicated that diesel fuels and nitrogen fertilizers are key factors in ozone layer depletion. Moreover, integrating precision agriculture techniques, such as variable rate application systems, can further mitigate the environmental impacts of fertilizer use. By tailoring fertilizer applications to the specific needs of the trees based on real-time data, farmers can achieve better nutrient management while reducing chemical inputs. **Fig 9** shows the environmental emissions of inputs in four final impact categories per ton of oranges produced using a fixed rate sprayer.

Direct emissions from the input’s consumption had the most environmental impacts on human health. Chemical fertilizers, including nitrogen, potassium and phosphate, had the most impacts on the ecosystem quality. Nitrogen fertilizer was the most effective factor on climate change. The total environmental impacts of producing one ton of oranges were 0.063 DALY and 123919.80 PDF*m^2^*yr for human health and ecosystem quality, respectively. The emissions from producing one ton of oranges were 422.86 kg of CO_2_ eq, and nitrogen fertilizer use caused the greatest environmental impact in the orange production process.

**Fig 10:**
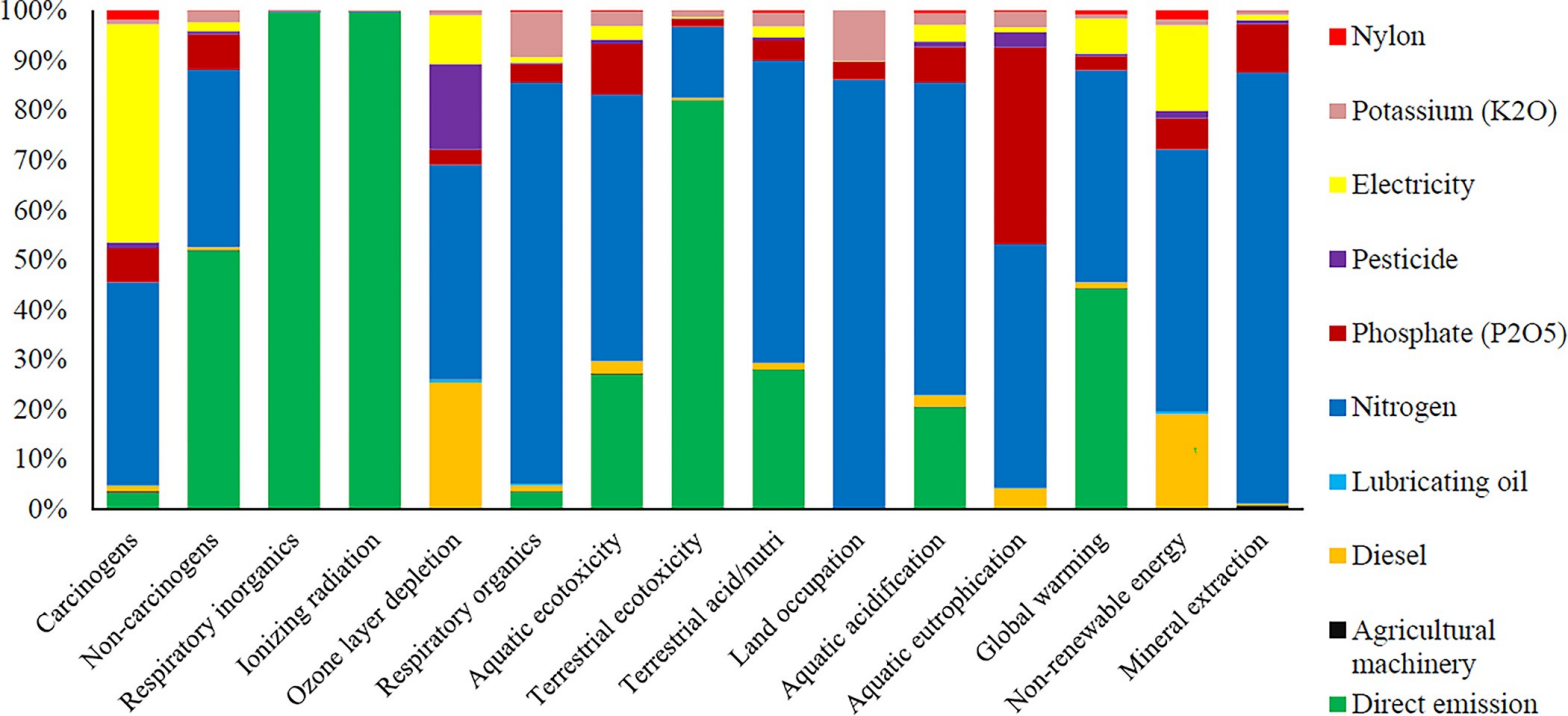
The results of the evaluation of environmental impacts in 15 intermediate impacts categories for using a variable rate sprayer.

**Fig 10** shows the intermediate effect indicators for producing one ton of oranges using a variable rate sprayer.

**Fig 11:**
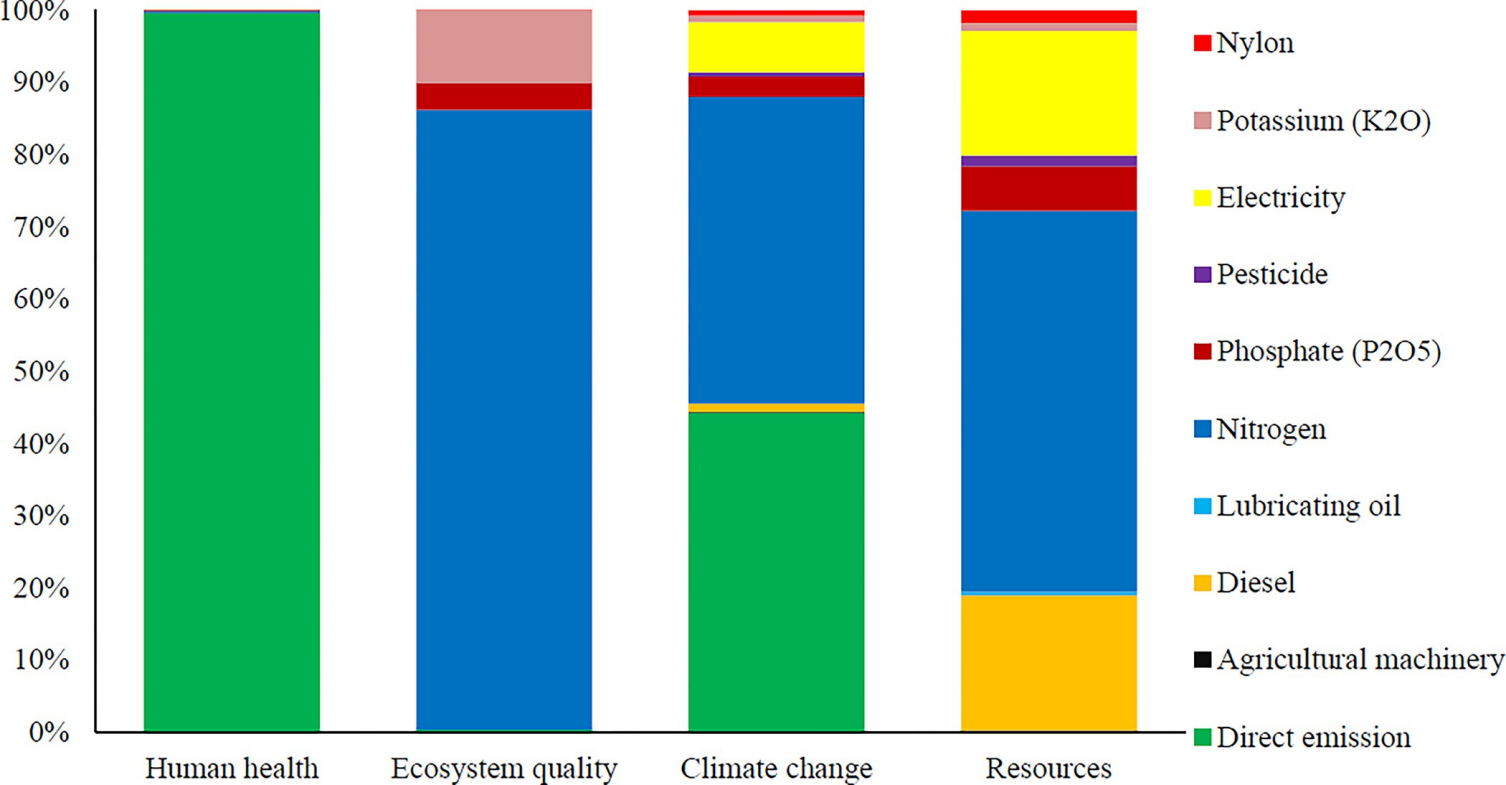
The results of environmental impact Assessment in 4 final impacts categories for using a variable rate sprayer.

In this scenario, all inputs such as nitrogen fertilizer, phosphate fertilizer, potassium fertilizer, etc were fixed and changed only the inputs affected by the variable rate sprayers. Variable rate sprayer reduced fuel consumption, pesticides consumption and some other inputs. It also increased the efficiency of the inputs use, as shown by its energy indicators. The environmental impact study showed that variable rate sprayers reduced environmental emissions compared to fixed rate sprayers. The GWP was 407.57 kg CO_2_ eq per ton of oranges produced in the variable rate spraying scenario. Nitrogen fertilizer and direct emissions from the production system had the most environmental impacts in most categories. **Fig 11** shows the environmental impacts in four final impact categories for variable rate sprayer in the orange production process.

Variable rate sprayer in orchard spraying reduced the environmental impacts compared to fixed rate sprayer. Direct emissions from the production system and nitrogen fertilizer had the most environmental impacts on human health with variable rate sprayer. Nitrogen fertilizer had the most impacts on the ecosystem quality.

**Fig 12:**
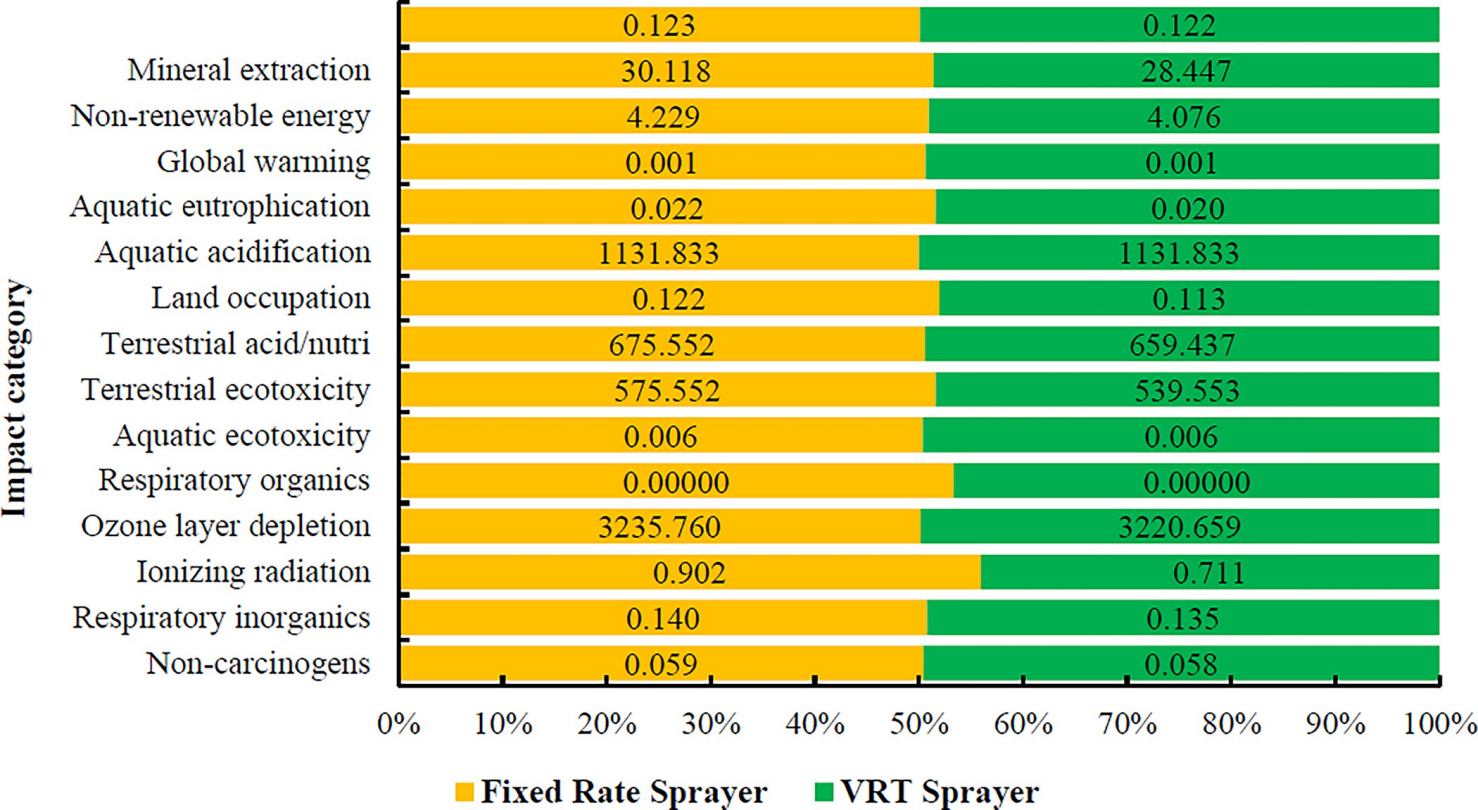
Comparison results of environmental impact assessment in 15 intermediate impact categories in two scenarios of using fixed and variable rate sprayers.

The total environmental impacts of producing one ton of oranges with variable rate sprayer were 0.049 DALY and 123905.87 PDF*m^2^*yr for human health and ecosystem quality, respectively. The emissions from producing one ton of oranges were 407.57 kg CO_2_ eq for climate change. **Fig 12** compares the intermediate impacts indicators for the orange production process under the two scenarios.

Variable rate spraying reduced environmental impacts compared to fixed rate spraying. The global warming potential decreased from 422.860 to 407.57 kg of carbon dioxide (kg CO_2_ eq) per ton of oranges produced with variable rate sprayer. Acidification of waters and respiratory inorganic substances also decreased from 2.178 to 2.041 kg SO_2_ eq and from 90.224 to 71.134 kg PM2.5 eq, respectively. Non-renewable resources decreased from 3011.79 to 2844.72 MG/TON with variable rate sprayer (**Table 10).**

**Table 10:** The results of environmental impact assessment in 15 intermediate impact categories in two scenarios of using fixed rate and variable rate sprayers in the production of one ton of oranges.

Impact category	Unit	Fixed Rate Sprayer	VRT Sprayer
Carcinogens	kg C_2_H_3_Cl eq	5.898	5.799
Non-carcinogens	kg C_2_H_3_Cl eq	13.969	13.537
Respiratory inorganics	kg PM2.5 eq	90.224	71.134
Ionizing radiation	Bq C-14 eq	323576.044	322065.852
Ozone layer depletion	kg CFC-11 eq	0.00003	0.00003
Respiratory organics	kg C_2_H_4_ eq	0.643	0.634
Aquatic ecotoxicity	kg TEG water	57555.162	53955.288
Terrestrial ecotoxicity	kg TEG soil	67555.234	65943.740
Terrestrial acid/nutri	kg SO_2_ eq	12.222	11.287
Land occupation	m2org.arable	113183.344	113183.313
Aquatic acidification	kg SO_2_ eq	2.178	2.041
Aquatic eutrophication	kg PO_4_ P-lim	0.066	0.065
Global warming	kg CO2 eq	422.860	407.573
Non-renewable energy	MJ primary	3011.790	2844.726
Mineral extraction	MJ surplus	12.272	12.215

A research evaluated the environmental performance of orange production in Mexico using the life cycle evaluation (cradle-to-gate) approach for two scenarios: organic and conventional production systems. The functional unit was one ton of oranges. The system boundaries included the environmental effects of orange cultivation, harvesting and inputs production. Orange production in the conventional system had more environmental impacts than the organic system in most categories, especially GWP, EP and AP, mainly due to fertilizer production and use. Upstream processes were the main environmental impacts of organic orange production [64].

The results of the study indicated that the use of variable rate sprayers significantly reduced environmental impacts across all four end impact categories. This reduction was primarily attributed to decreased consumption of chemical pesticides, diesel fuel, and agricultural machinery in scenarios utilizing variable rate sprayers. By optimizing the application of pesticides based on real-time data regarding crop needs and environmental conditions, these systems effectively minimize unnecessary chemical usage, leading to lower environmental emissions. In addition to mitigating environmental emissions, the implementation of variable rate sprayers also contributes to a reduction in input energy consumption and associated costs. This dual benefit is crucial for promoting sustainable agricultural practices. Lower energy consumption not only translates into cost savings for farmers but also reduces the overall carbon footprint of orange production. The latest researches proofed that if the necessary technologies and measures and proper management of inputs are not available and used, the environmental effects will be increased in the future [65] but by using appropriate technologies and improve the management, it is possible to optimize the consumption of resources and achieve the maximum productivity [22]. Optimal use of energy in agriculture, can reduces the environmental effects and make the achievement of sustainable agriculture [66]. It was stated in research that the low price of diesel fuel and the lack of incentive and punitive policies for producers with optimal consumption are the reasons for high consumption of diesel fuel in the process of production of agricultural products in Iran [67]. Also it was stated in a research that the small size of farms and the transportation of inputs by agricultural machinery on a small scale were are the reasons for the reduced efficiency of the use of inputs in cantaloupe production [68]. Therefore, the use of technologies such as variable spraying rate technology and other solutions such as land integration can lead to increased productivity and reduced environmental impacts.

**Fig 13:**
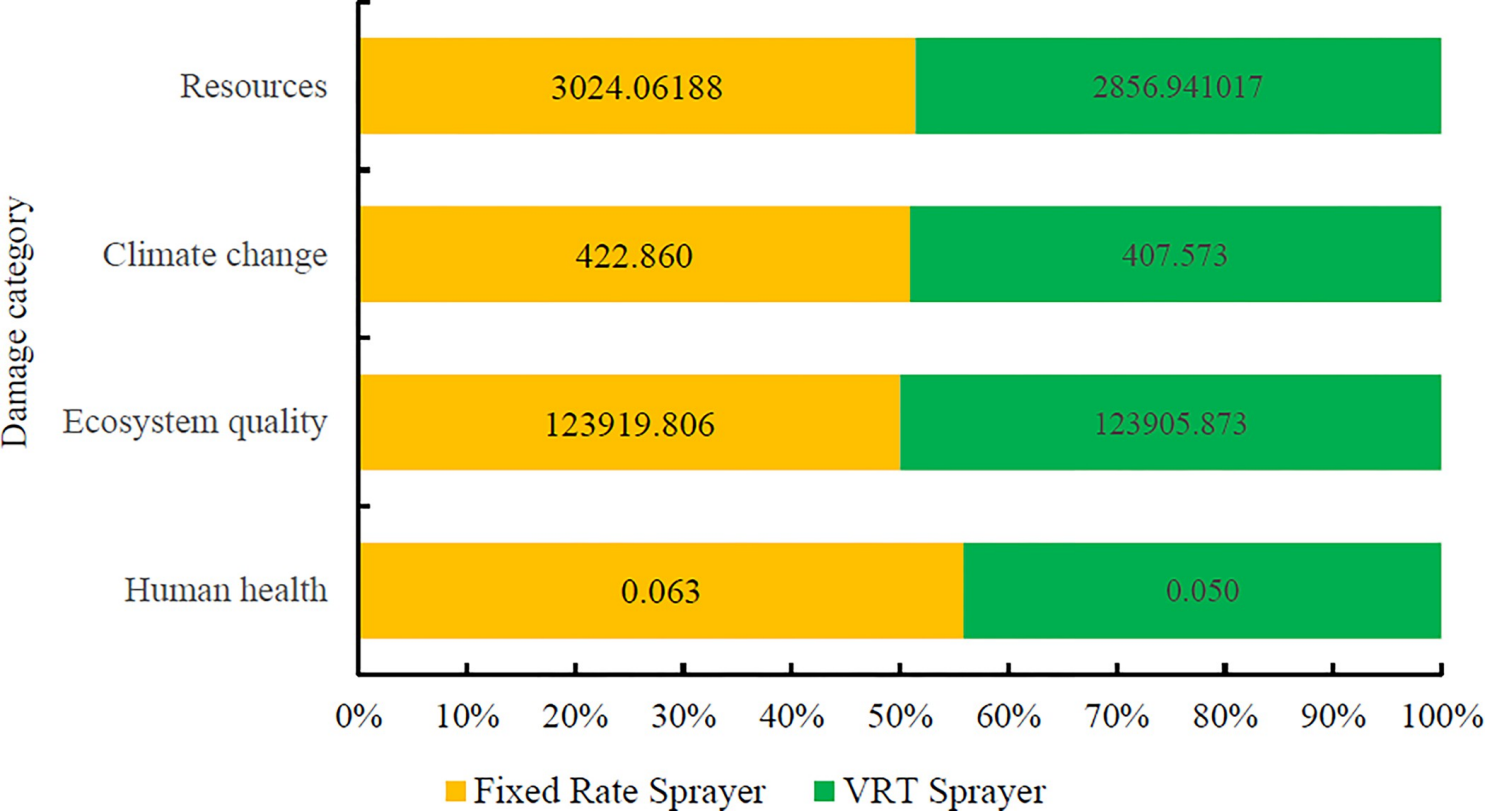
Comparison results of environmental impact assessment in 4 categories of final impact in two scenarios of using fixed rate and variable rate sprayers.

**Fig 13** showed the comparison of the final impacts indicators in the production of one ton of oranges under two scenarios of fixed rate and variable rate spray application.

## 4. Conclusions

The aim of this study is to develop and evaluate a fixed-rate orchard sprayer and compare it to a variable-rate orchard sprayer using different equipment. The application of variable rate spraying resulted in a significant reduction in pesticide sedimentation across all treatments when compared to fixed-rate spraying. Both the type of sprayer and the speed of spraying had a notable influence on pesticide deposition across different treatments. However, the interaction effect between these two factors was not statistically significant regarding pesticide deposition on trees and total pesticide consumption per hectare. The implementation of variable rate spraying improved energy efficiency from 0.594 to 0.650 and increased energy productivity from 0.313 to 0.342 kg MJ^-1^. Additionally, it enhanced net energy gain, shifting from -23,829.32 to -18,813.80. Variable rate spraying also mitigated environmental impacts compared to fixed-rate spraying; specifically, the global warming potential was reduced from 422.860 to 407.57 kg of CO₂ equivalent per ton of oranges produced with the variable rate sprayer. Furthermore, variable rate sprayers decreased environmental impacts across all four final impact categories. The reduction in the consumption of chemical pesticides, diesel fuel, and agricultural machinery associated with variable rate sprayers contributed significantly to lower environmental emissions.

## Supporting information

S1 Data (XLSX)

## Abbreviations

LCA: Life Cycle Assessment
SQI: Spray Quality Index
ADF: Abiotic Depletion for Fossil fuels
VMD: Volume Median Diameter
OLD: Ozone Layer Depletion
NMD: Number Median Diameter
FWE: Fresh Water aquatic Ecotoxicity
RGNIR: Red-Green-Near Infrared
MAE: Marine Aquatic Ecotoxicity
VRT: Variable Rate Technology
PO: Photochemical Oxidation
DE: Direct Energy
TE: Terrestrial Ecotoxicity
IE: Indirect Energy
AD: Abiotic Depletion
RE: Renewable Energy
AC: Acidification
NRE: Non-Renewable Energy
EU: Eutrophicatio
ER: Energy Ratio
GWP: Global Warming Potential
EP: Energy Productivity
HT: Human Toxicity
SE: Specific Energy
GHG: Greenhouse Gase
NEG: Net Energy Gain
